# Functional synergy and genomic linkage of glyphosate resistance traits in Canada fleabane

**DOI:** 10.1002/ps.70194

**Published:** 2025-09-04

**Authors:** Eric R Page, Sara L Martin, Sydney Meloche, Alyssa Thibodeau, Martin Laforest

**Affiliations:** ^1^ Harrow Research and Development Centre, Agriculture and Agri‐Food Canada Harrow Ontario Canada; ^2^ Ottawa Research and Development Centre, Agriculture and Agri‐Food Canada Ottawa Ontario Canada; ^3^ Saint‐Jean‐sur‐Richelieu Research and Development Centre, Agriculture and Agri‐Food Canada Saint‐Jean‐sur‐Richelieu Quebec Canada

**Keywords:** glyphosate resistance, target site resistance (TSR), non‐target site resistance (NTSR), synergy, genetic linkage, weed genomics

## Abstract

**BACKGROUND:**

Glyphosate resistance in *Conyza canadensis* (Canada fleabane) has been primarily attributed to non‐target‐site resistance (NTSR) mechanisms such as vacuolar sequestration, though these have not been formally elucidated. While a target‐site mutation at EPSPS2 (P106S) was recently identified, it failed to account for many resistant cases. These findings underscore the need to re‐evaluate the genetic basis of glyphosate resistance in this species.

**RESULTS:**

Using an F_2_ population derived from glyphosate‐resistant and susceptible biotypes, we disentangled the individual and combined effects of target‐site resistance (TSR) and NTSR. Dose–response phenotyping and genotyping revealed that NTSR conferred broad protection across a wide range of glyphosate doses, while TSR provided a more limited, dose‐dependent benefit. When both mechanisms were present, median lethal dose (LD_50_) values greatly exceeded additive expectations, indicating a synergistic interaction. Quantitative trait locus (QTL) mapping identified a major‐effect locus associated with NTSR on chromosome 4, with candidate genes linked to membrane transport and subcellular compartmentalization processes. Segregation distortion and recombination frequency estimates suggest moderate genetic linkage between TSR and NTSR loci, facilitating co‐inheritance of resistance alleles.

**CONCLUSION:**

This study provides the first explicit quantitative analysis of gene × gene interactions underlying herbicide resistance in *C. canadensis*. By disentangling TSR and NTSR, we show that single copies of the TSR and NTSR alleles confer approximately nine‐fold and seven‐fold glyphosate resistance, respectively. When combined, these mechanisms exhibit synergism, resulting in resistance levels that exceed additive LD_50_ expectations by more than two‐fold. Both TSR and NTSR loci have been mapped to chromosome 4, and moderate genomic linkage (~27% recombination) between them will likely contribute to the persistence and spread of high‐level resistance, even under low selection pressure. © 2025 His Majesty the King in Right of Canada. *Pest Management Science* published by John Wiley & Sons Ltd on behalf of Society of Chemical Industry. Reproduced with the permission of the Minister of Agriculture and Agri‐Food Canada.

## Graphical abstract

Stacking target‐site and non‐target‐site glyphosate resistance mechanisms in *Conyza canadensis* results in strong synergistic effects. This study quantifies gene × gene interactions and maps linked resistance loci, advancing our understanding of herbicide resistance genetics and informing management strategies.

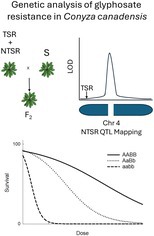

## 1 INTRODUCTION

Since its introduction in the 1970s, glyphosate has become a cornerstone of modern agriculture,^1^ enabling widespread adoption of herbicide‐tolerant crops and conservation tillage practices. It remains the most widely used herbicide globally. Glyphosate functions by inhibiting the chloroplastic, nuclear‐encoded enzyme 5‐enolypyruvylshikimate‐3‐phosphate synthase (EPSPS),^2^ disrupting the synthesis of aromatic amino acids. After absorption through the leaf cuticle and entry into the parenchymal cells,^3^, ^4^ it is translocated via the phloem to sink tissues, where it accumulates.^5^, ^6^ Inhibiting EPSPS reduces arogenate levels, which in turn disrupts the regulations of up‐stream enzymes, such as 7‐phospho 2‐dehydro 3‐deoxy‐d‐arabino‐heptulosonate (DAHP) synthase, ultimately compromising carbon flow to other essential pathways.^7^

In 2001, *Conyza canadensis* (L.) Cronquist *sensu lato* (= *Erigeron canadensis* L.) (horseweed, marestail, Canada fleabane) became the first annual dicotyledonous weed with reported resistance to glyphosate.^8^ Subsequent studies found no evidence of target‐site mutations in the EPSPS,^9^, ^10^, ^11^, ^12^, ^13^, ^14^ instead identifying non‐target‐site mechanisms as the primarily source of resistance. The most widely accepted non‐target‐site resistance (NTSR) mechanism involves vacuolar sequestration,^10^ where glyphosate is actively transported into vacuoles via adenosine triphosphate (ATP)‐dependent transporters, reducing its cytosolic and chloroplastic concentration and limiting its inhibitory effects on the shikimate pathway. This energy‐dependent process is associated with reduced translocation^11^ and likely works in concert with other NTSR mechanism, such as impaired translocation, collectively enabling *C. canadensis* to survive glyphosate exposure without EPSPS mutations.^10^, ^11^ Although vacuolar sequestration was first identified in 2010^10^ and is widely cited as the dominant resistance mechanism in this species, the specific genes responsible remain unknown.^15^, ^16^

The focus on NTSR mechanisms in glyphosate resistant *C. canadensis* persisted until 2018 when it was reported that a majority of Canadian glyphosate resistant *C. canadensis* biotypes possessed a proline to serine substitution at position 106 (P106S) of EPSPS2. This target site mutation has been widely reported to confer resistance to glyphosate in a number of other weed species^17^, ^18^, ^19^, ^20^, ^21^ but had never before been identified in *C. canadensis*. *Conyza canadensis* has three EPSPS genes (EPSPS1, EPSPS2, EPSPS3); EPSPS1 and EPSPS2 are shared with related species, while EPSPS3 likely arose from duplication of EPSPS2 but is now a pseudogene due to loss of function.^22^, ^23^ The similarity in sequence between these three EPSPS genes presents a challenge for designing polymerase chain reaction (PCR) primers and it is possible the lack of evidence of target‐site mutations in previous *C. canadensis* literature could be attributable to amplification of the wrong EPSPS gene. A recent study by Page *et al*.^24^ highlighted this exact occurrence in both *Ambrosia artemisiifolia* L. (common ragweed) and *Ambrosia trifida* L. (giant ragweed). Early reports of NTSR in these ragweed species overlooked target site mutations in EPSPS2 because of a lack of primer specificity. This issue was overcome with the recent publication of genomes for both ragweed species,^25^ emphasizing the need for species‐specific genomic data when elucidating molecular mechanism of herbicide resistance.^26^

The discovery of target‐site resistance (TSR) in Canadian biotypes of *C. canadensis* prompted a re‐evaluation of glyphosate resistant biotypes from the United States.^27^, ^28^ Having initially reported on high levels of glyphosate resistance in *C. canadensis* biotypes collected from Iowa and Ohio in 2015,^28^ Beres *et al*.^27^ re‐evaluated a subset of this initial collection and determined that, in hindsight, those expressing the highest levels of resistance (i.e., survival at 16 800 or 33 600 g acid equivalent (a.e.) glyphosate ha^−1^) possessed a P106S mutation that had been previously overlooked. This study marked the first documentation of target‐site mediated glyphosate resistance in *C. canadensis* within the United States and initiated a discussion on the relative contributions of target and non‐target site mechanisms to the observed levels of glyphosate resistance in this species. While the studies by Page *et al*.^29^ and Beres *et al*.^27^ linked target‐site mediated resistance to extreme levels of glyphosate resistance, the authors acknowledged the possibility that these biotypes also possessed non‐target‐site mechanisms.^27^, ^29^ It has been previously suggested that, relative to TSR for other active ingredients, mutations in EPSPS confer lower levels of resistance to glyphosate than do non‐target‐site mechanisms.^30^ To date, however, no study has directly quantified the individual or combined contributions of TSR and NTSR mechanisms to glyphosate resistance in *C. canadensis*.

The objective of this research is to determine whether TSR biotypes of *C. canadensis* also possess NTSR mechanisms to glyphosate. To achieve this, resistant and susceptible parents were crossed to generate a segregating F_2_ population. Survivors were then genotyped to confirm the presence or absence of the P106S target‐site mutation. For individuals lacking the P106S mutation, loci associated with NTSR were mapped using a genotype‐by‐sequencing (GBS) approach to identify if one or more regions in the genome were linked to resistance. From this analysis, a molecular marker for NTSR was generated and subsequently used to examine the relative levels of glyphosate resistance biotypes with TSR alone, biotypes with NTSR alone, and biotypes where both mechanisms co‐occur. By analyzing these groups, this study clarifies the individual and combined contributions of target‐site and non‐target‐site mechanisms to glyphosate resistance in *C. canadensis*, providing a comprehensive understanding of how these mechanisms interact to enhance resistance levels.

## 2 MATERIALS AND METHODS

### 2.1 Plant material

Two biotypes of *C. canadensis*, one glyphosate‐resistant (PR) and one susceptible (PS), were selected as parental lines for seed increase and subsequent experiments. These biotypes were among the 98 previously characterized by Page *et al*.^29^ Seeds of PR and PS were used to conduct seed increases during which seedlings of PR were screened with potassium salt of glyphosate at a dose of 900 g a.e. ha^−1^ (Roundup WeatherMAX with Transorb 2 Technology, 540 g a.e. L^−1^; Bayer CropScience Canada, Mississauga, Canada). Seedlings were grown in a glasshouse at the Harrow Research and Development Center (HRDC) under a 16 h:8 h photoperiod and a of 25 °C/20 °C thermoperiod. Herbicide was applied to *C. canadensis* seedlings (~7 cm diameter) using an automated spray chamber equipped with a TeeJet 8002E even flat‐fan nozzle (TeeJet Technologies, Glendale Heights, IL, USA), calibrated to deliver 333.3 L ha^−1^ at 30 psi. The nozzle was positioned 50 cm above the target, with a spray area of 0.81 m^2^, and the boom moved at a constant speed of 2.7 km h^−1^, ensuring uniform and consistent herbicide coverage. Survivors were grown to maturity and self‐pollination was ensured by enclosing flowering plants in a bag of pollination fabric (DelStar Technologies Inc, Austin, TX, USA) and isolating individuals in separate glasshouse compartments, where possible. A seed increase for PS was similarly conducted, but individuals of this biotype were not screened with glyphosate prior to self‐pollination.

### 2.2 Crossing

Seed from self‐pollinated PR and PS were used for all crosses. Seedlings of both biotypes were propagated under glasshouse conditions previously described and those of PR were screened with glyphosate at 900 g a.e. ha^−1^ (Supporting Information Fig. S1). Then, 10–14 days later, seedlings of both biotypes were placed into cold storage for 4 to 6 weeks at 4 °C to meet any vernalization requirements for the transition to reproductive growth (i.e., bolting). Once removed from cold storage, surviving seedlings were transplanted into larger pots and were left to grow to the reproductive stage in glasshouses under the same conditions as described earlier.

Leaf tissue was collected from each of the prospective parental plants before crossing began. EPSPS2 specific primers were used to amplify a 994‐bp region around and including the expected single nucleotide polymorphism (SNP) at P106.^29^ DNA was extracted from approximately 20 mg of that leaf tissue using a Macherey‐Nagel NucleoSpin Plant II kits (Macherey‐Nagel Inc., Bethlehem, PA, USA) following the manufacturer's protocol. The forward primer had a sequence of 5′‐TACATAGTGAGGTGCAAGGT‐3′, and the reverse primer was 5′‐TGTAGGAGGATGAAGCAGAC‐3′. Primers were synthesized by Integrated DNA Technologies (Coralville, IA, USA). Eluted DNA was amplified by PCR with the following reaction conditions: an initial denaturation at 94 °C for 3 min, 35 cycles of 94 °C for 30 s, 56 °C for 30 s for annealing, 72 °C for 1 min, followed by a final extension at 72 °C for 10 min. PCR products were visualized on a 2.5% agarose gel containing 5% nucleic acid staining solution (RedSafe, Froggabio Toronto, Canada). Following PCR, the samples were cleaned for sequencing using GenepHlow Gel/PCR kit (Geneaid Biotech, New Taipei City, Taiwan) according to the provided protocol. Sanger sequencing of the PCR products was carried out by the London Regional Genomics Center (Robarts Research Institute, London, ON, Canada) using the same primers from the PCR amplification. Alignment to the *C. canadensis* EPSPS2 reference sequence (Genbank accession #AY545667.1) was performed using the Sequencher software (version 5.4.6; Gene Codes, Ann Arbor, MI, USA) and analyzed for the presence of the P106S target‐site mutation. Only individuals shown to be homozygous for proline (PS) or serine (PR) at position 106 of EPSPS2 were used in subsequent crosses.

The crossing methods used in the current study closely follow those outlined by Zelaya *et al*.^31^ and Hickmott *et al*.^32^ In brief, each cross was initiated by selecting pairs of individuals from the desired set of parental biotypes whose floral initiation was in close synchrony. Capitula (inflorescence) of *C. canadensis* contain pistillate ray and perfect disk florets (flowers) and controlled crossing between individuals was thus achieved by the removal of the disk florets (i.e., emasculation) using forceps and the transfer of the desired pollen to the ray florets. To assess the efficiency of our emasculations, a capitulum was selected at the top of each plant to serve as a negative control (i.e., was emasculated and covered to prevent outcrossing). Capitula used for crossing were selected at random and emasculated when they reached the appropriate stage of development (see figure 1 of Hickmott *et al*.^32^). The remaining ray florets were cross pollinated once a day, every day for 7 to 10 days until the capitula closed, indicating the onset of seed maturation. Reciprocal pollen transfer was achieved by emasculating donor capitula from an individual of the desired parental biotype and brushing these mature perfect disk florets on the ray florets of the emasculated recipient capitulum.

When mature, F_1_ achenes (hereafter referred to as seeds) were harvested they were immediately placed in an incubator to germinate. As *C. canadensis* seeds exhibit little to no primary dormancy at maturity,^33^ they germinate readily under favorable conditions. Controlled crosses typically yield up to 20 F_1_ seeds,^32^ which were placed into Petri dishes lined with moist blue blotter paper (steel‐blue germination blotters; Anchor Paper, St Paul, MN, USA), and incubated under the following conditions: a 25 °C/10 °C day/night thermoperiod, 60% relative humidity and a 14‐h photoperiod. Once germinated, seedlings were transplanted and grown in a glasshouse under the same conditions as previously described. All seedlings were genotyped for P106S (as describe earlier) and only those demonstrated to be heterozygous were retained. All heterozygous F_1_ individuals were once again cold acclimated to accelerate bolting. The progeny from separate crosses were segregated by glasshouse compartment and all individuals were covered with DelNet pollination bags (DelStar Technologies Inc.) prior to flowering to ensure self‐pollination and facilitate the collection of the F_2_ seed. Seeds from each plant were kept as separate F_2_ families.

### 2.3 Inheritance of glyphosate resistance

The F_2_ progeny arising from the reciprocal crosses among the parental biotypes were screened at two glyphosate doses: 3600 and 7200 g a.e. ha^−1^. Seedlings of the F_2_ generation and their parental biotypes were propagated in glasshouse plug flats under the conditions previously described. Once the rosettes reached 7 cm in diameter, experimental units were created by transplanting rosettes as plugs in a new flat that contained an individual plant of each parent and an F_2_ individual produced from each of the reciprocal crosses between these parental biotypes (i.e., six F_2_ rosettes + two parental rosettes per tray). Survival was recorded at 14 days after application (DAA), using a strict criterion: any plant displaying visible green tissue was considered alive. This criterion was applied consistently across all portions of the study to ensure uniformity in phenotyping and downstream analyses. At each discriminating dose, segregation ratios in the F_2_ generation were analyzed by *χ* ^2^ test.^34^ The *χ* ^2^ test for homogeneity was performed to determine whether segregation data could be combined across families.

Individuals surviving a dose of 7200 g a.e. ha^−1^ of glyphosate were genotyped for the P106S target site mutation using Sanger sequencing. The segregation ratio in these surviving individuals was again analyzed by *χ* ^2^ test. Twenty‐seven individuals, genotyped as homozygous for proline at position 106, were retained and self‐pollinated to produce NTSR F_2_S_1_ lines. Seedlings of these lines were then produced for use in dose–response assays. When seedlings had reached the 7 cm diameter (rosette) growth stage they were sprayed with the potassium salt of glyphosate at doses of 0, 225, 450, 900, 1800, 3600, 7200, 14 400, 28 800 and 57 600 g a.e. ha^−1^ using the methods previously described. The experimental design for the dose–response trial was a randomized complete block with five replicates and was repeated twice in time. Seedlings of the parental biotypes, PR and PS, were included in each replicate as controls. Plants were assessed 14 DAA, scored for injury and the aboveground biomass was harvested, dried in a forced air dryer and weighed.

Dose response of parental biotypes and F_2_S_1_ families were conducted as completely randomized design with ten replications, and two repetitions in time. Survival and dried aboveground biomass were measured and analyzed using dose–response models in SAS version 9.4 (SAS Institute, Cary, NC, USA). Survival data (binary: alive or dead) were analyzed using the PROC NLMIXED procedure, fitting a two‐parameter log‐logistic model of the form:

$$f\left(x\right)=\frac{1}{1+{\left(\frac{x}{{\mathrm{LD}}_{50}}\right)}^{b}}$$

where *f*(*x*) is the probability of survival at dose *x*, LD_50_ is the dose at which 50% mortality occurs, and *b* is the slope parameter. Aboveground biomass was expressed as a percentage of the untreated control and analyzed using PROC NLIN. A separate three‐parameter log‐logistic model was used, with the response scaled by an upper asymptote *d*, according to:

$$f\left(x\right)=\frac{d}{1+{\left(\frac{x}{{\mathrm{GR}}_{50}}\right)}^{b}}$$

where *f*(*x*) is the percent biomass at dose *x*, GR_50_ is the dose causing a 50% reduction in biomass, *d* is the upper asymptote, and *b* is the slope.

### 2.4 Selection and screening F_2_S_1_ individuals of the mapping population

Results from the F_2_S_1_ dose response described in Section 3.3 were used as the basis for selecting an F_2_S_1_ line for subsequent GBS. The primary selection criteria was segregating survivorship at 14 400 g a.e. ha^−1^ glyphosate. Subsequently 570 individuals of the selected F_2_S_1_ line were propagated to spray size as described previously and screened with a glyphosate dose of 14 400 g a.e. ha^−1^. Simultaneously, 220 individuals of each of PR and PS were also propagated and screened. Leaf tissue was collected prior to glyphosate application, lyophilized and DNA was extracted following the procedures as described earlier. Overall survivorship of the F_2_S_1_ line was 73% and a subset of 470 individuals were selected and included on five 96‐well plates for GBS (i.e., 301 alive, 169 dead). Included on each plate were one well of PR and one of PS DNA produced through the extraction and pooling of ten individuals of each biotype.

### 2.5 Genotype by sequencing (GBS)

Libraries for triple digest GBS (3D‐GBS) were prepared using the procedure described by Poland *et al*.^35^ with modifications at the Plateforme d'Analyses Génomiques of the Institut de Biologie Intégrative et des Systèmes (IBIS, Université Laval, Québec, Canada). The modifications consist of the following: three restriction enzymes (*Pst*I/*Nsi*I/*Msp*I) were used instead of the *Pst*I/*Msp*I combination and a blue Pippin (SAGE Sciences, Beverly, ME, USA) was used to size libraries before PCR amplification (elution set between 50 and 65 min, on a 2% gel). Plate barcodes were added according to the procedure described in Colston‐Nepali *et al*.^36^ Libraries were loaded on an Element Biosciences AVITI instrument (Element Biosciences, San Diego, CA, USA) using a 2 × 75 Cloudbreak FS kit to generate SE150 reads following manufacturer's instructions.

### 2.6 Genotyping by sequencing data analysis

The sequence reads obtained from genotyping by sequencing were demultiplexed using EA Utils^37^ fastq‐multx, trimmed using fastp^38^ version 0.24.0 and then aligned to the *C. canadensis* genome^23^ using BWA^39^ version 0.7.17‐r1188. Alignment files were sorted and indexed with samtools^40^ version 1.10 and polymorphisms were identified using bcftools^41^ version 1.14. The parameters for SNP filtering with vcftools^42^ were max‐missing 0.5, mac 3, minQ 30, minDP 3 and individuals with more than 50% missing markers (missing‐indv); SNP not meeting these criteria were removed. Subsequent analyses were performed in R^43^ version 4.3.3 (Angel Food Cake) with packages vcfR,^44^ R/qtl^45^ and R/qtl2.^46^ LOD (logarithm of the odds) thresholds were calculated with 1000 permutations for the Haley–Knott regression and the linear mixed model (LMM) using either the standard kinship matrix or the leave one chromosome out (LOCO) method in qtl2/R. Gene annotation was performed by performing a Diamond Blast^47^ search against the National Center for Biotechnology Information (NCBI) Genbank^48^ non‐redundant database NR and InterProScan^49^ database 103.0 with OmicsBox version 3.4.0.^50^

### 2.7 Genotyping TSR and NTSR traits in an F_2_ dose response

A SNP was identified through GBS that was strongly associated with non‐target site glyphosate resistance. Primers were designed to amplify a 591 bp region of chromosome 4 containing this SNP. The forward primer had a sequence of 5′‐CACGTGTAACACCAACCAAAG‐3′, and the reverse primer was 5′‐ACACACACAGCTTCTCTCAC‐3′. Five hundred seedlings from the F_2_ generation were propagated to the 7 cm diameter (rosette) growth stage and were sprayed with the potassium salt of glyphosate at doses of 0, 225, 450, 900, 1800, 3600, 7200, 14 400, 28 800 and 57 600 g a.e. ha^−1^ using the methods previously described. Plants were assessed 14 DAA, scored for injury and the aboveground biomass was harvested, dried in a forced air dryer and weighed. DNA was extracted from approximately 20 mg of that leaf tissue using a Macherey‐Nagel NucleoSpin Plant II kits following the manufacturer's protocol. Primers for the NTSR SNP, as well as those for genotyping P106S described in Section 3.2, were synthesized by Integrated DNA Technologies. Eluted DNA was amplified by PCR using the same reaction conditions outlined in Section 3.2 for both sets of primers. Sanger sequencing of the PCR products was carried out by the London Regional Genomics Center using the same primers from the PCR amplification. Alignment to the TSR and NTSR loci were performed using the Sequencher software. Only individuals with complete phenotypic and genotypic data (i.e., survival and biomass data, and clean sequence reads for both TSR and NTSR loci) were included in subsequent analyses.

The observed genotype frequencies were analyzed using a *χ* ^2^ goodness‐of‐fit test to assess conformity to expected Mendelian ratios for a two‐gene dihybrid cross. Expected proportions for the nine F_2_ genotypic classes (AABB, AABb, AAbb, AaBB, AaBb, Aabb, aaBB, aaBb, aabb) were based on the independent assortment of two unlinked loci (A and B, representing the NTSR and TSR loci, respectively), assuming Mendelian segregation (expected ratios: 1/16, 2/16, 1/16, 2/16, 4/16, 2/16, 1/16, 2/16, 1/16, respectively). The PROC FREQ procedure was used to test for deviation from expected ratios.

To test for genetic linkage between loci A and B, genotypes were grouped into four phenotypic classes based on the presence or absence of dominant alleles: A_B_, A_bb, aaB_, and aabb. Frequencies for each class were entered into a separate dataset and analyzed using a contingency *χ* ^2^ test in PROC FREQ with the CHISQ option. Recombination frequency (RF) was estimated using a weighted gamete‐based method. Each genotype was assigned a recombinant gamete count based on classical expectations from a dihybrid cross under coupling‐phase linkage. Genotypes AAbb and aaBB, which can only arise from two recombinant gametes, were assigned a weight of 2 recombinant gametes per individual. Genotypes AABb, AaBB, Aabb, aaBb, which can arise from one recombinant and one parental gamete, were assigned 1 recombinant gamete per individual. Genotypes AABB, AaBb, and aabb, which contain only non‐recombinant gametes, were assigned 0. The total number of recombinant gametes was summed across all genotypes, and RF was calculated as:

$$\mathrm{RF}=\frac{\text{Total number of recombinant gametes}}{\text{Total gametes}}$$

This approach allows inclusion of partially informative genotypes while maintaining proportional contributions to RF.

The effects of genotype at the NTSR and TSR loci along with herbicide dose and their interactions, were modeled using the full set of F_2_ genotypic combinations (AA, Aa, aa and BB, Bb, bb). This design enabled the detection of additive and dominant effects across a continuous herbicide dose gradient (0–57 600 g active ingredient (a.i.) ha^−1^). Plant survival was treated as a binary outcome and analyzed using generalized linear mixed models (GLMMs) with a binomial distribution and logit link, implemented in PROC GLIMMIX. Fixed effects included herbicide dose (continuous), genotype at NTSR and TSR loci, and their two‐way interactions with dose. Predicted survival probabilities were generated using PROC PLM, applied to a reference dataset containing all genotype combinations across the full dose range. These model‐based predictions were used to construct genotype‐specific dose–response curves. LD_50_ values were estimated from logistic models using the ESTIMATE and ILINK statements in PROC GLIMMIX. Confidence intervals were calculated using the delta method.

Final plant biomass was analyzed as a continuous response variable using a LMM implemented in PROC MIXED, assuming a normal distribution and identity link function. To meet model assumptions, non‐zero biomass values were log‐transformed prior to analysis. Fixed effects included herbicide dose (modeled as a continuous covariate), genotype at NTSR and TSR loci, and their two‐way interactions with dose. Least‐squares means for significant effects were back‐transformed from the log scale to generate interpretable estimates.

We initially fit full factorial models for both survival (binary logistic regression) and biomass (linear mixed‐effects model using log‐transformed biomass) to assess the effects of herbicide dose and genotype at two loci (NTSR and TSR). These models included all main effects, two‐way interactions (Dose × NTSR, Dose × TSR, NTSR × TSR), and the three‐way interaction (Dose × NTSR × TSR). However, because genotyping was conducted after herbicide application, genotypes were not evenly distributed across doses. This imbalance, compounded by deviations from expected genotypic ratios due to genetic linkage, complicated model fitting by genotype. Including all higher‐order interactions further contributed to overfitting, as indicated by inflated standard errors, non‐significant terms, and poor model interpretability. Residual diagnostics also revealed increased variance and violations of model assumptions. To improve model stability and clarity, we systematically removed non‐informative interactions. Final models retained only significant main effects and key two‐way interactions, capturing most of the variation while maintaining good fit. Model simplification was guided by Akaike information criterion (AIC) comparisons, residual analysis, and the principle of parsimony.

## 3 RESULTS

### 3.1 Response of parental biotypes to glyphosate

**Figure 1:**
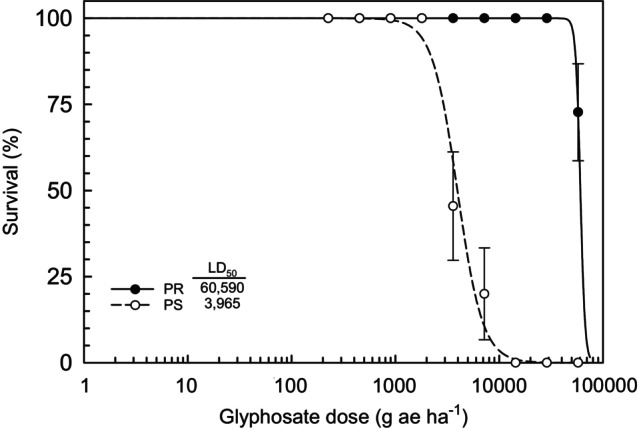
Survival of *Conyza canadensis* parental resistant (PR, close circles) and parental susceptible (PS, open circles) biotypes, as influenced by glyphosate dose. A two‐parameter log‐logistic equation (*f*(*x*) = 1/(1 + (*x*/LD_50_)^*b*^) was fit to PR (*b* = 21.4, LD_50_ = 60 306), and PS (*b* = 3.2, LD_50_ = 4212).

**Figure 2:**
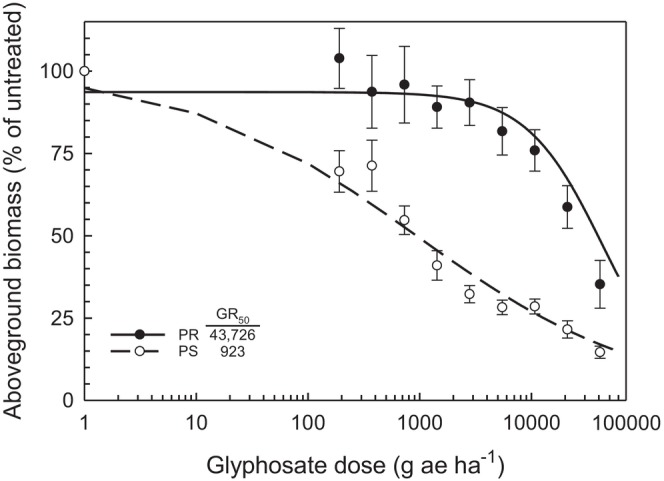
Aboveground biomass of *Conyza canadensis* parental resistant (PR, close circles) and parental susceptible (PS, open circles) biotypes, as influenced by glyphosate dose. A three‐parameter log‐logistic equation (*f*(*x*) = 1/(1 + (*x*/GR_50_)^*b*^) was fit to PR (*d* = 97.9, *b* = 1.08, GR_50_ = 38 223), and PS (*d* = 100.8, *b* = 0.48, GR_50_ = 1240).

The characterization of PR and PS as glyphosate resistant and susceptible, respectively, was confirmed through dose response. Results indicate that the LD_50_ ranged from 60 590 g a.e. ha^−1^ for the PR to 3965 g a.e. ha^−1^ for PS (Fig. 1). Based on these results, the PR biotype exhibited a resistance factor of 15‐fold relative to the PS. The response of aboveground biomass to glyphosate dose was similar to that measured for survival (Fig. 2) with the GR_50_ ranging from 43 726 g a.e. ha^−1^ for the PR to 923 g a.e. ha^−1^ for the PS.

### 3.2 Inheritance of glyphosate resistance

The segregation of the F_2_ generation produced from a cross of the PR and PS biotypes was evaluated at two glyphosate doses (Table 1). At a dose of 3600 g a.e. ha^−1^, F_2_ survival approached 83% (149/179) and there was no mortality in the PR biotype. There was, however, survival in the PS with 3 out of 30 individuals alive 14 DAA. The *χ* ^2^ test of the pooled F_2_ indicated that these results deviated from the 3:1 ratio expected under the assumption of monogenic inheritance of the glyphosate resistance trait (Table 1). At 7200 g a.e. ha^−1^, all PS individuals were controlled and there was no mortality in the PR. Survival in the F_2_ progeny approached 68% (122/180) and the *χ* ^2^ test of the pooled F_2_ indicated that these results also deviated from the expected 3:1 ratio (Table 1). When the 122 F_2_ individuals surviving 7200 g a.e. ha^−1^ were genotyped for P106S, 27 were identified as homozygous for proline indicating that their survival was not attributable to target site mutation but rather to other non‐target site mechanism(s) (Table 2).

**Table 1:** Survival of parental biotypes and F_2_ families of *Conyza canadensis* at two glyphosate doses

	Treated	Survived	Expected	Survival (%)	*χ* ^*2*^	df	Probability
*3600 g a.e. ha* ^*−1*^							
Susceptible	30	3		10			
Resistant	30	30		100			
Total F_2_	179	149	134	83	6.482	1	0.011
Test of heterogeneity					4.927	5	0.425
*7200 g a.e. ha* ^*−1*^							
Susceptible	30	0		0			
Resistant	30	30		100			
Total F_2_	180	122	135	68	5.007	1	0.025
Test of heterogeneity					8.979	5	0.110

*Note*: Observed survival was compared to expected Mendelian segregation ratios under the assumption of monogenic inheritance of a single resistance gene.

**Table 2:** Distribution of the P106S target site mutation in EPSPS2 of the F_2_ individuals surviving a glyphosate dose of 7200 g a.e. ha^−1^

F_2_ family	P106	Heterozygous	106S	Total	*χ* ^2^	df	Probability
1	3	9	4	16	0.38	1	0.540
2	2	10	8	20	3.60	1	0.058
3	7	13	0	20	6.70	1	0.010
4	4	13	4	21	1.19	1	0.275
5	6	13	4	23	0.74	1	0.390
6	5	8	9	22	3.09	1	0.079
Total F_2_	27	66	29	122	0.885	1	0.347
Test of heterogeneity					14.81	5	0.011

*Note*: Observed survival was compared to expected Mendelian segregation ratios under the assumption of monogenic inheritance with codominant expression, predicting a 1:2:1 distribution among genotypic classes.

### 3.3 Characterization of non‐target site resistant F_2_S_1_ individuals

**Figure 3:**
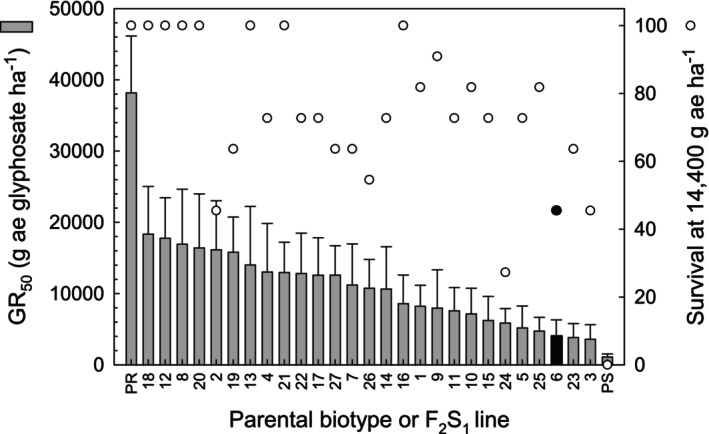
Biological parameters for selecting a *Conyza canadensis* F_2_S_1_ line for use in genotype‐by‐sequencing (GBS). Glyphosate dose at GR_50_ and survival 14 days after the application of a glyphosate dose of 14 400 g a.e. ha^−1^ were used to select line 6 for subsequent GBS analysis.

Twenty‐seven F_2_S_1_ lines were produced from F_2_ individuals that had both survived 7200 g a.e. ha^−1^ and were genotyped as homozygous for proline at position 106 of EPSPS2 (Table 2). Dose response of these F_2_S_1_ indicated that the GR_50_ of these NTSR lines ranged from 18 436 to 3728 g a.e. ha^−1^ (Fig. 3). These values represent 50% and 90% reductions, respectively, relative to the GR_50_ of the PR biotype, which possesses both TSR and NTSR mechanisms. Survival of the F_2_S_1_ at a glyphosate dose of 14 400 g a.e. ha^−1^ ranged from 45% to 100% and this benchmark was used a metric for selecting a line for use in GBS. Based on the results presented in Fig. 3, F_2_S_1_–6 was selected because its survival at this glyphosate dose (i.e., 45%) indicated that NTSR was still segregating in this line.

### 3.4 Identification of QTLs linked to glyphosate resistance

**Figure 4:**
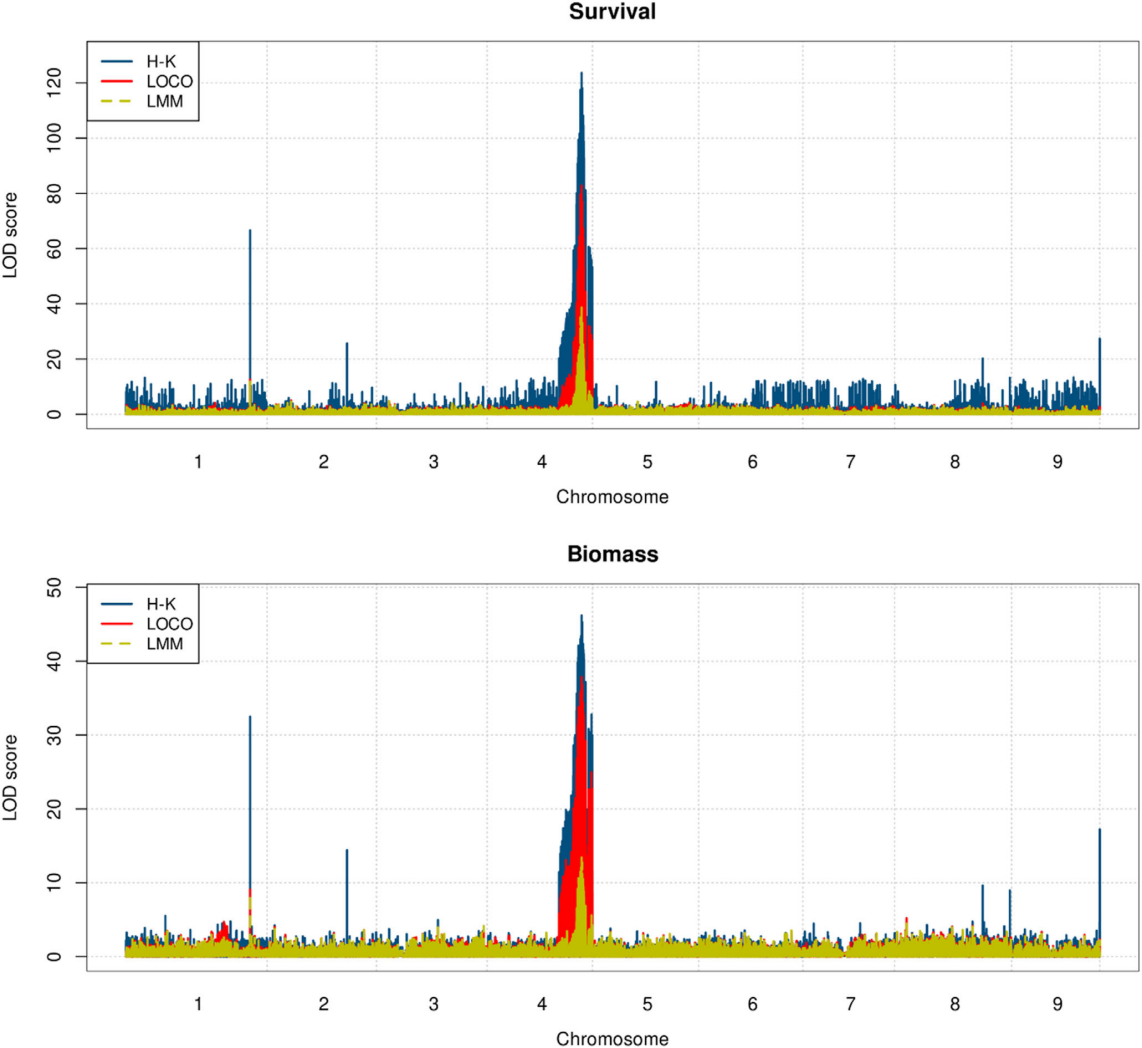
Quantitative trait locus (QTL) mapping results for two traits: survival (top panel) and biomass (bottom panel). The *y*‐axis shows LOD (logarithm of the odds) scores, representing the strength of association between genetic markers and phenotypic variation. The *x*‐axis corresponds to genomic positions across chromosomes 1–9. Three statistical models were used for QTL detection: Haley–Knott regression (H‐K, blue), linear mixed model (LMM, purple), and the leave‐one‐chromosome‐out approach (LOCO, green dashed line).

**Figure 5:**
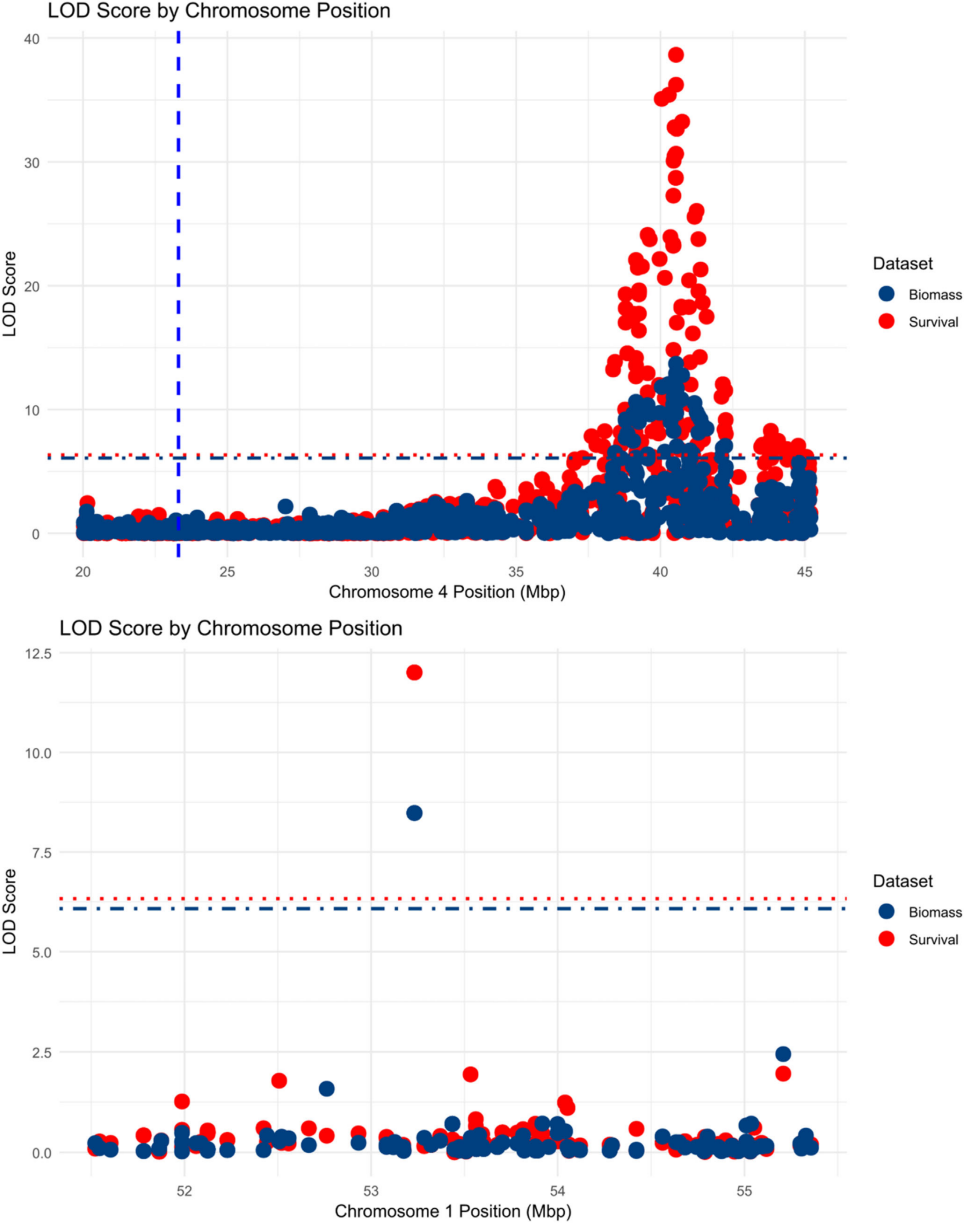
Zoomed‐in view of quantitative trait locus (QTL) mapping results for chromosome 4 (top) and chromosome 1 (bottom), showing logarithm of the odds (LOD) scores for survival (blue) and biomass (red). The *y*‐axis represents LOD scores, and the *x*‐axis indicates physical genomic positions in megabase pairs (Mbp). Each point represents a genetic marker. Horizontal dashed lines indicate the genome‐wide significance thresholds for each trait, while the vertical dashed line on chromosome 4 marks the position for EPSPS2.

Sequencing data from 470 individuals lead to the identification of 212 419 SNP. After filtering, the number of individuals and the number of SNP were reduced to 455 and 21 121 respectively. Haley–Knott regression and the LMM using either the standard kinship matrix or the LOCO method identified a locus with LOD scores well above the significance thresholds for both survivorship and biomass. The calculated thresholds for the association with biomass were 6.23, 6.08 and 6.22 for the Haley–Knott regression and the LMM using either the standard kinship matrix or the LOCO method, respectively. For survival, these values were 5.83, 6.33 and 6.23, respectively. Several Haley–Knott quantitative trait locus (QTL) are above the thresholds for both survival and biomass phenotypes. The LMM using either the standard kinship matrix or the LOCO method reduces the number of QTL to two, one located around positions 53 232 008 to 53 232 368 on chromosome 1, and a second major QTL on chromosome 4, with its peak at position 40 537 862 (Table 3 and Fig. 4). A closer look at the QTL on chromosome 1 (Fig. 5 and Supporting Information Table S1), reveals that there are only four SNPs located between positions 53 231 981 and 53 232 022, a region of the genome with no annotated gene. The QTL on chromosome 4, however, is much larger spanning more than five megabases (~37–42.3 Mbp) in the genome (Fig. 5 and Table S1) with the peak of the QTL located at position 40 537 862. This region contains 581 genes and the SNP at the peak of this QTL is located in the 3′ UTR of gene g20801. These genes were annotated and the results are listed in Table S2.

**Table 3:** Logarithm of odds (LOD) values of significant quantitative trait locus (QTL) peaks associated to survival and biomass traits with different methods to adjust for kinship

Traits	Chr	Peak position	LOD (H‐K)	LOD (LMM)	LOD (LOCO)
Survival	1	53 232 368	66.61	12.00	12.60
	2	34 277 241	25.64		
	3	35 927 898	11.16		
	4	40 537 862	123.63	38.65	82.82
	5	27 284 174	11.74		
	6	28 331 746	12.19		
	7	25 827 338	12.77		
	8	37 783 561	20.17		
	9	37 817 634	27.36		
Biomass	1	53 232 002	32.49	8.48	9.09
	2	34 277 241	14.41		
	4	40 537 862	46.19	13.70	37.86
	8	37 783 561	9.63		
	9	37 817 282	17.22		

*Note*: Chr, chromosome; H‐K, Haley–Knott; LMM, linear mixed model; LOCO, leave one chromosome out.

### 3.5 Genotyping TSR and NTSR traits in an F_2_ dose response

A total of 475 F_2_ individuals from an AABB × aabb cross were genotyped into nine classes based on the NTSR and TSR loci (Table 4). Results indicated that genotypic frequencies deviated significantly from the expected Mendelian 1:2:1:2:4:2:1:2:1 dihybrid ratio (*χ* ^2^ = 57.09, df = 8, *P* < 0.0001). The intermediate heterozygous class (AaBb) was notably overrepresented, while some homozygous classes, including AAbb and aaBB, were underrepresented. Linkage analysis revealed significant deviation from independent assortment between loci A (NTSR) and B (TSR) (*χ* ^2^ = 24.08, df = 1, *P* < 0.0001), with a *φ* coefficient of 0.2252, indicating a moderate degree of genetic linkage. Based on the distribution of recombinant and parental genotypes, RF was estimated at 27.3%, suggesting that crossover events occurred in roughly one‐third of gametes.

**Table 4:** Observed and expected genotype at the target site resistance (TSR) and non‐target site resistance (NTSR) loci (A and B, respectively) in 475 F_2_ individuals arising from a parental cross of AABB × aabb

Genotype	Observed	Expected (no linkage)	Recombinant gametes per individual	Total number of recombinant gametes	Total number of gametes
AABB	42	30	0	0	84
AABb	42	59	1	42	84
AAbb	12	30	2	24	24
AaBB	52	59	1	52	104
AaBb	144	119	0	0	288
Aabb	60	59	1	60	120
aaBB	11	30	2	22	22
aaBb	59	59	1	59	118
aabb	53	30	0	0	106
Total				259	950
Recombination frequency (%)					27.3

Biomass accumulation in response to glyphosate was significantly influenced by genotype at the NTSR locus (*P* = 0.0081, Table 5), with a clear additive trend: aa (0.068 g plant^−1^) < Aa (0.08 g plant^−1^) < and AA (0.10 g plant^−1^). This pattern indicates each additional NTSR allele incrementally increases biomass accumulation, consistent with additive gene action. A marginally significant interaction with dose was also detected (*P* = 0.0411), suggesting some divergence between AA and Aa genotypes at higher doses (data not presented). In contrast, the TSR locus showed no significant effect on biomass (*P* = 0.7507) and did not interact with glyphosate dose, indicating that it had no meaningful contribution to post‐treatment biomass accumulation.

**Table 5:** Effect of genotype at the target site resistance (TSR) and non‐target site resistance (NTSR) loci and their interaction with glyphosate dose on aboveground biomass and survival 14 days after application in the F_2_ generation

Fixed effect	NDF (numerator degrees of freedom)	DDF (denominator degrees of freedom)	Biomass	Survival
			*P*‐value	
Dose	1	416	<0.0001	<0.0001
NTSR	2	416	0.0081	0.0268
TSR	2	416	0.7507	0.1821
Dose × NTSR	2	416	0.0411	0.2603
Dose × TSR	2	416	0.6791	0.0083

Survival in response to glyphosate was strongly influenced by genotype at the NTSR locus (*P* = 0.0268; Table 5). In contrast to biomass results, survival was also significantly affected by TSR genotype in a dose‐dependent manner (*P* = 0.0083). At the NTSR locus, survival followed an additive trend: individuals with the aa genotype showed the lowest survival (0.54), followed by Aa (0.75) and AA (0.85). Because the NTSR × Dose interaction was not significant, this rank order remained consistent across the full range of glyphosate doses. The TSR locus also showed an additive effect, with survival increasing from bb to Bb to BB, but the strength of this effect varied with dose. At lower doses (< 10 000 g a.e. ha^−1^), survival differences among genotypes were minimal (Fig. S2). These differences became more pronounced at intermediate doses (10 000–30 000 g a.e. ha^−1^), with BB individuals exhibiting the highest resistance. However, as glyphosate doses exceeded ~45 000 g a.e. ha^−1^, survival across TSR genotypes began to converge, suggesting that the protective effect of TSR diminished at extreme doses. This divergence in response patterns likely reflects underlying mechanistic differences. NTSR, potentially mediated by reduced glyphosate translocation, may provide consistent protection by limiting herbicide accumulation in sensitive tissues. In contrast, TSR relies on an altered EPSPS target site, which may offer only dose‐dependent protection that is eventually overcome as intracellular glyphosate concentrations rise. NDF: numerator degrees of freedom; DDF: denominator degrees of freedom.

**Figure 6:**
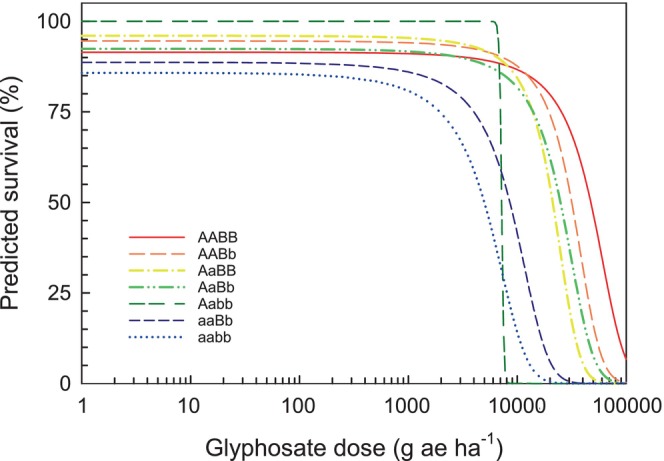
Dose–response curves illustrating glyphosate resistance across eight genotypic classes of *Conyza canadensis* defined by combinations of target‐site resistance (TSR, B locus) and non‐target‐site resistance (NTSR, A locus) alleles. A two‐parameter logistic equation [*f*(*x*) = 1/(1 + exp(−(*b*_0_ + *b*_1_*x)))], where *b*_0_ is the intercept and *b*_1_ is the slope (% survival/g a.e. ha^−1^), was fit to AABB (*b*_0_ = 2.3716, *b*_1_ = −0.00005), AABb (*b*_0_ = 2.8599, *b*_1_ = −0.00009), AaBB (*b*_0_ = 3.1837, *b*_1_ = −0.00015), AaBb (*b*_0_ = 2.5001, *b*_1_ = −0.00010), Aabb (*b*_0_ = 44.460 *b*_1_ = −0.00617), aaBb (*b*_0_ = 2.0586, *b*_1_ = −0.00024) and aabb (*b*_0_ = 1.7989, *b*_1_ = −0.00036). Curves represent model‐predicted survival probabilities across glyphosate doses. Median lethal dose (LD_50_) values, calculated as –*b*_0_/b_1_, were estimated from the models: AABB = 45 143 g a.e. ha^−1^, AABb = 32 822 g a.e. ha^−1^, AaBB = 20 886 g a.e. ha^−1^, AaBb = 24 937 g a.e. ha^−1^, Aabb = 7201 g a.e. ha^−1^, aaBb = 8675 g a.e. ha^−1^ and aabb = 4932 g a.e. ha^−1^. Estimates for AAbb and aaBB are not shown due to low sample sizes and poor model convergence.

LD_50_ values varied markedly by genotype, illustrating the independent and combined contributions of the NTSR (A) and TSR (B) loci to glyphosate resistance (Fig. 6). The susceptible baseline (aabb) had the lowest LD_50_ at 4932 g a.e. ha^−1^. Single‐mechanism genotypes, Aabb and aaBb, showed moderate resistance with LD_50_ values of 7201 and 8675 g a.e. ha^−1^, respectively. Resistance increased substantially when both mechanisms were present: AaBb, AaBB, AABb, and AABB genotypes exhibited LD_50_ values from 20 886 to 45 143 g a.e. ha^−1^, with AABB showing the highest resistance. The underrepresentation of AAbb and aaBB in the F_2_ contributed to model fitting issues and unstable LD_50_ estimates for these genotypes (data not presented). The LD_50_ for AAbb was exceptionally high, with wide confidence intervals and a non‐significant *t*‐value, while aaBB also showed high variability and borderline statistical significance. These patterns reflect reduced sample sizes and diminished statistical power for these homozygous classes.

To evaluate the interaction between resistance loci, Colby's equation^51^ was applied to estimate the expected additive LD_50_ for the AaBb genotype. This value, calculated as Aabb + aaBb − aabb, was 10 944 g a.e. ha^−1^. In contrast, the observed LD_50_ for AaBb was 24 937 g a.e. ha^−1^, over twice the expected value. Because the expected LD_50_ (10 944 g a.e. ha^−1^) lies well below the 95% confidence interval of the observed LD_50_ for the AaBb genotype (18203–31 671 g a.e. ha^−1^), the results provide strong statistical support for a synergistic interaction between the A and B loci.

## 4 DISCUSSION

### 4.1 Mapping non‐target site glyphosate resistance in *C. canadensis*

Results of this study demonstrate that extreme glyphosate resistance in *C. canadensis* biotypes arises from the combined action of TSR and NTSR mechanisms, which interact synergistically to amplify resistance beyond the level conferred by either mechanism alone. GBS analysis identified a major QTL associated with non‐target site glyphosate resistance on the fourth chromosome of *C. canadensis*, spanning over five megabases (~37–42.3 Mbp). Importantly, the survival and biomass phenotypes both strongly aligned with this QTL, reinforcing its role in resistance. While EPSPS2 and EPSPS3 are also located on chromosome 4, they fall outside the identified QTL region, with EPSPS2 positioned approximately 17 Mb from the QTL peak at 40 537 862 bp.

Our QTL mapping results reveal exceptionally strong genetic associations with non‐target‐site glyphosate resistance, most notably at the major locus on chromosome 4. This region yielded LOD scores as high as 123.63 using Haley–Knott regression and 82.82 with the LOCO method, values that far exceed those typically reported in the quantitative genetics literature. In weed science, the application of QTL mapping to NTSR has been relatively limited until recently, primarily due to a lack of genomic resources.^26^ This is beginning to change with advances in sequencing technologies and projects like the International Weed Genomics Consortium. In a recent example, Werle *et al*.^52^ identified distinct NTSR mechanisms to dicamba and 2,4‐D in *Amaranthus tuberculatus* (Moq.) Sauer, reporting a maximum LOD of 18.2 for dicamba resistance. While lower than the LOD reported in our study, this value is well within the range commonly observed in other pesticide resistance studies, such as those on pyrethroid resistance in the *Leptinotarsa decemlineata* (Say)^53^ and *Anopheles funestus* ^54^ for example.

The strength of our results also stands out when compared to QTL mapping in plant breeding, where yield traits have been the most frequent targets. Yield‐related QTLs in crops like corn (*Zea mays* L.), rice (*Oryza sativa* L.), and soybean (*Glycine max* (L.) Merr.) typically show LOD scores between 3 and 15, reflecting their highly polygenic and environmentally sensitive nature.^55^, ^56^, ^57^ The much higher LOD scores observed in our study likely reflects a simpler genetic basis for herbicide resistance, possibly involving one or a few major‐effect loci under strong directional selection from herbicide application. This makes such loci more readily detectable compared to those governing more complex, polygenic traits like yield or disease resistance.

The identified QTL region contains 581 annotated genes based on gene ontology (GO) information (Table S2). These genes span diverse functional categories, with the most common GO terms including protein binding, membrane localization, ATP binding, nucleic acid binding, and functions related to proteolysis. Among these, the gene located at the peak of the QTL on chromosome 4 is annotated as a CASP‐like protein (g20801.t1). This gene may contribute to structural or signaling functions in the plant cell wall, potentially influencing the movement or compartmentalization of xenobiotics.^58^, ^59^ Casparian strip‐associated proteins are best known for their role in the root endodermis, where they form a lignified apoplastic barrier that regulates solute uptake. These structures have been shown to restrict the movement of xenobiotics into the vascular system. Although endodermis‐like layers containing Casparian strips have been identified in aerial tissues such as stems and petioles of various herbaceous species, their physiological function in these tissues remains under‐explored.^60^ Notably, it is unclear whether Casparian strips in aerial organs play any role in limiting the vascular entry of foliar‐applied herbicides like glyphosate, which depend on phloem loading and transport to reach target tissues such as the shoot apical meristem. Other genes of interest in the QTL region include two WAT1‐related proteins (g20782.t1 and g20783.t1), which are known to localize to the tonoplast and function as auxin transporters or vacuolar transport facilitators.^61^ These WAT1‐like proteins have been implicated in vacuolar sequestration mechanisms, suggesting a plausible role in glyphosate resistance by mediating the compartmentalization of glyphosate into vacuoles and thus reducing its cytosolic availability and toxicity.^10^, ^61^, ^62^ These genes are located 18 and 17 genes upstream (~188 kb upstream), respectively, from the CASP‐like gene (g20801.t1) at the peak of the QTL, placing them in close physical proximity and supporting their potential functional relevance to the resistance phenotype.

### 4.2 Synergism defines the interaction of target and non‐target site glyphosate resistance mechanisms in *C. canadensis*

It has long been recognized that herbicide resistance mechanisms can interact within plants to enhance resistance to specific active ingredients. Several studies have demonstrated such interactions at the whole‐plant level (e.g., Ghanizadeh *et al*.^63^ and Han *et al*.^64^), providing important insights into the expression of stacked resistance traits. However, these studies often leave key questions unanswered, particularly regarding the mechanistic interplay between TSR and NTSR traits, the influence of gene dosage, and the evolutionary implications of genetic linkage between resistance loci.^65^

Our study offers a detailed and quantitative assessment of these dynamics at the level of individual resistance alleles. The relative contributions of TSR and NTSR to glyphosate resistance were clearly distinguishable. NTSR (locus A) conferred broad and robust protection across a wide range of glyphosate doses, with high survival persisting even beyond 40 000 g a.e. ha^−1^. In contrast, TSR (locus B) provided more limited and dose‐dependent protection, with its effects diminishing at higher glyphosate doses. This hierarchy in efficacy aligns with theoretical expectations that different resistance mechanisms vary in strength and selective advantage.

One of the most compelling findings of our study was the strong synergistic effect between TSR and NTSR mechanisms. Individuals carrying both resistance loci (e.g., AaBb) exhibited LD_50_ values more than double the additive expectation calculated using Colby's equation,^51^ a widely used method for assessing herbicide interactions. The predicted additive LD_50_ (10 944 g a.e. ha^−1^) fell well outside the 95% confidence interval of the observed value (24 937 g a.e. ha^−1^), providing robust statistical support for synergism. These findings are consistent with the conceptual framework of Bliss independence,^66^ which assumes that two factors act independently to affect a common outcome; in this case, survival following glyphosate treatment. Under this framework, the observed survival response exceeding the predicted value strongly indicates positive interaction between the mechanisms. This pattern mirrors observations from insecticide resistance research, where synergistic effects commonly occur between homozygous resistance loci and are also seen in heterozygous combinations.^67^ Such synergism is believed to result from complementary modes of action, for example, one mechanism reducing effective herbicide concentrations via sequestration, while the other lowers target site sensitivity, together amplifying the resistance phenotype beyond additive expectations.

Beyond their functional interaction, our data indicate that the TSR and NTSR loci are physically linked on the same chromosome, separated by approximately 17 Mb with a RF of 27.3%. While this recombination rate is not low enough to indicate tight linkage, it is below the 50% expected for unlinked loci, suggesting moderate linkage between these resistance mechanisms. This genetic proximity is further supported by segregation distortion in the F_2_ population, particularly the underrepresentation of recombinant genotypes (e.g., AAbb and aaBB), which deviates from Mendelian expectations. This linkage has important implications: moderate linkage between TSR and NTSR loci can promote the co‐inheritance of resistance alleles, helping to preserve synergistic combinations that confer enhanced glyphosate resistance, even under moderate selection pressure, such as low herbicide doses. Under strong selection pressure, such linkage may also accelerate the fixation of multi‐mechanism resistance haplotypes and hinder the breakdown of resistance through recombination, thereby complicating efforts to manage or reverse resistance evolution in weed populations.^67^, ^68^, ^69^

## 5 CONCLUSIONS

To our knowledge, this is the first herbicide resistance study to explicitly and quantitatively assess gene × gene interactions across a full range of genotypic combinations. By estimating LD_50_ values for all major genotype classes, we found strong evidence that TSR and NTSR interact synergistically in *C. canadensis* producing much higher resistance levels than expected under an additive model. This demonstrates that the combined presence of both mechanisms substantially enhances glyphosate resistance, advancing our understanding of how resistance traits interact at the functional level.

Beyond phenotypic interactions, we identified a strongly associated major‐effect QTL linked to glyphosate resistance, likely representing the NTSR locus. This region exhibited exceptional statistical support and yielded a shortlist of candidate genes potentially involved in detoxification or transport processes. Furthermore, evidence of physical linkage between TSR and NTSR loci, located on the same chromosome with reduced recombination, suggests that these traits may be inherited together, promoting the stable transmission of multi‐mechanism resistance in natural populations.

More broadly, this study provides a blueprint for analyzing complex herbicide resistance traits in the era of weed genomics. By integrating classical inheritance models, genotypic interaction analyses, and high‐resolution QTL mapping, we offer a multifaceted approach to understanding resistance evolution. These findings highlight the importance of accounting for both genetic interactions and genomic context in resistance modeling and management, emphasizing the need for integrated strategies to mitigate the spread of robust, multi‐locus herbicide resistance.

## CONFLICT OF INTEREST

The authors declare no conflict of interest.

## AUTHOR CONTRIBUTIONS

Eric Page: conceptualization, formal analysis, funding acquisition (Co‐PI), investigation, project administration, supervision, validation, visualization, and writing original draft preparation. Sara Martin: conceptualization, data curation, formal analysis, investigation, visualization and writing original draft preparation. Sydney Meloche: investigation. Alyssa Thibodeau: investigation. Martin Laforest: conceptualization, data curation, formal analysis, funding acquisition (Co‐PI), supervision, validation, visualization, and writing original draft preparation.

## Supporting information


**Figure S1.** Crossing and screening scheme used to dissect glyphosate resistance mechanisms in *Conyza canadensis*. Susceptible (PS) and resistant (PR) parental biotypes were crossed to produce F_1_ hybrids, which were self‐pollinated to generate F_2_ progeny. F_2_ individuals were screened at two glyphosate doses (3600 and 7200 g a.e. ha^−1^), and survivors at the higher dose were genotyped for the EPSPS2 P106S mutation to distinguish target‐site resistance (TSR) from non‐target‐site resistance (NTSR). Homozygous non‐mutant survivors were advanced to F_2_S_1_ lines for dose–response phenotyping and selection of segregating families for QTL mapping. This approach enabled the independent and combined evaluation of TSR and NTSR mechanisms.

**Figure S2.** Dose‐dependent survival across TSR genotypes (bb, Bb, BB) in response to glyphosate application.

**Table S1.** Chromosomal position of filtered SNP with LOD (logarithm of the odds) scores for the survival and biomass phenotypes.

**Table S2.** Names of transcripts for the genes located in the QTL region, with their annotations obtained with Interproscan. Annotations include length of the sequence, the number of matches in the database, the score of the alignment, the mean similarity of the sequence to its hits in a BLAST search, InterPro IDs, InterPro GO IDs and InterPro GO Names.

## Data Availability

The data that support the findings of this study are available from the corresponding author upon reasonable request.
