# Supplementary material for: Functional synergy and genomic linkage of glyphosate resistance traits in Canada fleabane
Source: Pest Manag Sci. 2025 Sep 4;82(1):305–18. doi: 10.1002/ps.70194 (PMC12713706; doi:10.1002/ps.70194)
Supplement: Supplementary file 1 — Figure S1. Crossing and screening scheme used to dissect glyphosate resistance mechanisms in Conyza canadensis. Susceptible (PS) and resistant (PR) parental biotypes were crossed to produce F1 hybrids, which were self‐pollinated to generate F2 progeny. F2 individuals were screened at two glyphosate doses (3600 and 7200 g a.e. ha−1), and survivors at the higher dose were genotyped for the EPSPS2 P106S mutation to distinguish target‐site resistance (TSR) from non‐target‐site resistance (NTSR). Homozygous non‐mutant survivors were advanced to F2S1 lines for dose–response phenotyping and selection of segregating families for QTL mapping. This approach enabled the independent and combined evaluation of TSR and NTSR mechanisms. [file PS-82-305-s003.docx]

**Figure S1.** Crossing and screening scheme used to dissect glyphosate resistance mechanisms in Conyza canadensis. Susceptible (PS) and resistant (PR) parental biotypes were crossed to produce F₁ hybrids, which were self-pollinated to generate F₂ progeny. F₂ individuals were screened at two glyphosate doses (3600 and 7200 g ae ha⁻¹), and survivors at the higher dose were genotyped for the EPSPS2 P106S mutation to distinguish target-site resistance (TSR) from non-target-site resistance (NTSR). Homozygous non-mutant survivors were advanced to F₂S₁ lines for dose–response phenotyping and selection of segregating families for QTL mapping. This approach enabled the independent and combined evaluation of TSR and NTSR mechanisms.
