# Supplementary material for: Functional synergy and genomic linkage of glyphosate resistance traits in Canada fleabane
Source: Pest Manag Sci. 2025 Sep 4;82(1):305–18. doi: 10.1002/ps.70194 (PMC12713706; doi:10.1002/ps.70194)
Supplement: Supplementary file 2 — Figure S2. Dose‐dependent survival across TSR genotypes (bb, Bb, BB) in response to glyphosate application. [file PS-82-305-s001.docx]

**Figure S2.** Dose-dependent survival across TSR genotypes (bb, Bb, BB) in response to glyphosate application*.*
